# Multiscale forward electromagnetic model of uterine contractions during pregnancy

**DOI:** 10.1186/1756-6649-12-4

**Published:** 2012-11-05

**Authors:** Patricio S La Rosa, Hari Eswaran, Hubert Preissl, Arye Nehorai

**Affiliations:** 1 Department of Internal Medicine-General Medical Sciences Division, Washington University School of Medicine, Saint Louis, Missouri 63110, USA; 2 Department of Obstetrics and Gynecology, University of Arkansas for Medical Sciences, Little Rock, Arkansas 72205, USA; 3 MEG-Center, University of Tübingen, Tübingen, Germany; 4 Department of Electrical and Systems Engineering, Washington University, Saint Louis, Missouri 63130, USA

## Abstract

**Background:**

Analyzing and monitoring uterine contractions during pregnancy is relevant to the field of reproductive health assessment. Its clinical importance is grounded in the need to reliably predict the onset of labor at term and pre-term. Preterm births can cause health problems or even be fatal for the fetus. Currently, there are no objective methods for consistently predicting the onset of labor based on sensing of the mechanical or electrophysiological aspects of uterine contractions. Therefore, modeling uterine contractions could help to better interpret such measurements and to develop more accurate methods for predicting labor. In this work, we develop a multiscale forward electromagnetic model of myometrial contractions during pregnancy. In particular, we introduce a model of myometrial current source densities and compute its magnetic field and action potential at the abdominal surface, using Maxwell’s equations and a four-compartment volume conductor geometry. To model the current source density at the myometrium we use a bidomain approach. We consider a modified version of the Fitzhugh-Nagumo (FHN) equation for modeling ionic currents in each myocyte, assuming a plateau-type transmembrane potential, and we incorporate the anisotropic nature of the uterus by designing conductivity-tensor fields.

**Results:**

We illustrate our modeling approach considering a spherical uterus and one pacemaker located in the fundus. We obtained a travelling transmembrane potential depolarizing from −56 mV to −16 mV and an average potential in the plateau area of −25 mV with a duration, before hyperpolarization, of 35 s, which is a good approximation with respect to the average recorded transmembrane potentials at term reported in the technical literature. Similarly, the percentage of myometrial cells contracting as a function of time had the same symmetric properties and duration as the intrauterine pressure waveforms of a pregnant human myometrium at term.

**Conclusions:**

We introduced a multiscale modeling approach of uterine contractions which allows for incorporating electrophysiological and anatomical knowledge of the myometrium jointly. Our results are in good agreement with the values reported in the experimental technical literature, and these are potentially important as a tool for helping in the characterization of contractions and for predicting labor using magnetomyography (MMG) and electromyography (EMG).

## Background

Modeling the myometrium contractility during pregnancy is of clinical importance, since it can aid in understanding the mechanism of labor, and, thus, it can help in monitoring the health of both the fetus and the mother. The occurrence of labor is in general accompanied by the appearance of periodic contractions which increase the intrauterine pressure to the point that cervix dilatation is manifested [1]. However, from clinical experiences, not all uterine contractions are efficient, i.e., lead to labor. Term labor is expected to occur after the 37*th* week of pregnancy, but in the last decade the incidence of preterm labor has increased significantly (12% of all births). Preterm birth can cause health problems or even be fatal for the fetus if it happens too early, and, at the same time, it imposes significant financial burdens on health care systems [2]. Therefore, it becomes critical to better understand the mechanism of bioreproduction which, as a consequence, would allow for the development of more effective forms of therapy that might help to predict labor and control the occurrence of labor. In the following we will discuss briefly the uterine microanatomy, previous contractions models, and our modeling approach.

### Uterine microanatomy

**Figure 1:**
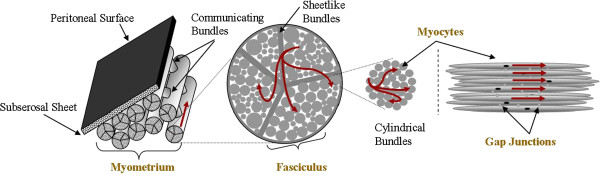
**Diagram of microanatomy of pregnant human myometrium **[4]**.** Red lines represent current flows.

The adult uterus is a thick walled, hollow, muscular organ formed by three layers: the external serous perimetrium, the myometrium, and the inner mucous endometrium [1]. The non-pregnant uterus wall thickness is approximately 15 mm to 20 mm, and during pregnancy the uterine wall becomes thin with values at the 39th week of pregnancy ranging from 7.4 ±1.8 mm at the low anterior wall (lower segment) at the bladder interface, to 10.06 ± 1.9 mm at the posterior wall [3]. The myometrium is responsible for contractions, and it is formed by fasciculi which are comprised of sheet-like and cylindrical bundles of myocytes embedded in a connective tissue matrix [4]. The myocytes in a cylindrical bundle contract, thus shortening the smooth tissue and increasing uterus wall tension, hence increasing the intrauterine pressure. Figure 1 illustrates the microanatomy of the pregnant human myometrium.

The uterine microanatomy is consistent with action potential propagation [4]: (i) myocytes are densely packed within a bundle, (ii) bundles are contiguous within a fasciculus, and (iii) fasciculi are contiguous via communicating bridges formed with myocytes. In addition, the uterine changes during gestation are accompanied by the formation of gap junctions, which are one of the mechanisms for transmitting of contractile activity from cell to cell in a coordinated manner [1,4]. The structure of the fasiculata within the uterus has not yet been well defined, but generally it makes the propagation of the action potential anisotropic [5,6].

### Uterine contraction models

Uterine contractions can be described by their mechanical and electrophysiological aspects. A mechanical contraction is manifested as a result of the excitation as well as the propagation of electrical activities in the uterine muscle, and appears in the form of an intrauterine pressure increase. Existing models approach the problem separately at the organ level [7-12] or at the cellular level [13-17].

At the organ level, the models presented in [7-11] focus on predicting the contractile forces that closely resemble clinical measurements of normal intrauterine pressure during contractions in labor, and, more recently, the action potential propagation. In [7], the authors assume that the uterus is a hollow ovoid formed by discrete contractile elements that propagate electrical impulses, generate tension, and have defined contracting and refractory periods. The envisioned mechanism for intercellular communication is based on action potential propagation, which is simulated by using a discrete state model for each cell. In [8], the authors revisit the model developed in [7] and perform multiple experiments with different uterine shapes, cell numbers, and initial distributions of active and resting cells. In [9], the author uses a discrete state model for combining action potential propagation and intercellular calcium wave propagation, two mechanisms of intercellular communication. However, in [7-9], mathematical and physical descriptions of the models are not provided. In [11], the trajectories of growth and myometrial tension at the onset of labor are modelled using a statistical modeling approach. The authors model the trajectories of uterine wall thickness, volume, and tension as functions of gestational age using a prolate ellipsoid method, intrauterine pressure results from the literature, and ultrasound measurements of the shape of the uterus collected on 320 subjects. Regarding the electrical activity of uterine contractions, a model of myometrial action potential propagation as measured by surface electrohysterography is proposed in [10]. The authors develop a myometrium skin conduction model consisting of four parrallel layers (myometrium,abdomen,fat, and skin), and model the action potential using a Gamma probability distribution. This model assumes that the electrical conducitivity of the myometrium is isotropic and that the abdominal curvature is negligible, thus making it suitable to model the electrical potential in a limited area of the abdominal surface. Recently, in [12], the authors model the electrical propagation of action potential using a 3-dimensional myometrium for different initial conditions and stimuli. The authors use a reaction-difussion equation coupled with a Fizhugh-Nagumo model of ionic currents and consider a homogeneous isotropic myometrium.

At the cellular level, the models focus on predicting the changes of ionic concentrations in the intracellular and extracellular media during a contraction, and, as a consequence, on modeling the transmembrane potential evolution of a myocyte as a function of time. In [13,14] a model is developed to simulate the complete process of a single myometrial smooth muscle contraction, which is initiated by depolarization. The model is based on the electrophysiological properties of a myocyte and on the cellular mechanisms that relate the rise in concentration levels of intracellular ion calcium **Ca**^2 + ^to stress production. In [17], the authors model all known individual ionic currents of myometrial smooth muscle close to labor and combined them into a mathematical model of myometrial action potential generation. The model is shown to successfully mimic several recordings of spontaneous AP and force in uterine smooth muscle.

**Figure 2:**
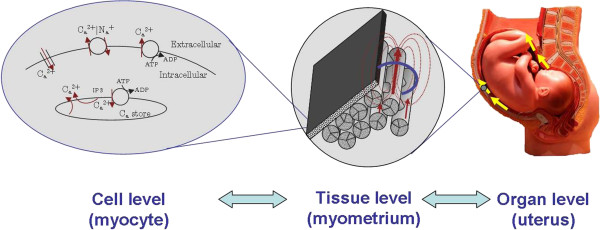
Illustration of the proposed modeling approach.

In this work, we propose a multiscale forward electromagnetic model of human myometrial contractions during pregnancy that jointly takes into account electrophysiological and anatomical knowledge at the cellular, tissue, and organ levels. Our model aims to help in the characterization of contractions and the prediction of labor using MMG [18] and EMG [19]. Here, we extend our partial results presented in [20]. Figure 2 illustrates the different levels considered in our modeling approach. In particular, our approach is twofold: first, we model the current source density at the myometrium, using models of myocyte electrophysiological activity and anisotropic conductivity, and second, we solve the forward electromagnetic problem, specifically, we compute the magnetic field and the action potential at the abdominal surface generated by the myometrial current-source density using Maxwell’s equations subject to a volume conductor geometry. To model the current source density at the myometrium we propose to apply a bidomain approach. The bidomain equations are a set of reaction-diffusion equations derived first for modeling the current sources of the myocardium as a function of the cardiac-myocyte transmembrane potential, and these equations proved to be a successful approach to study the functioning of the heart [21,22]. The diffusion part of the equations governs the spatial evolution of the transmembrane potential, and the reaction part is given by the local ionic current cell dynamics. Here we introduce a modified version of the FitzHugh-Nagumo (FHN) equation for modeling ionic currents in each myocyte. Though FHN does not consider explicitly the **Ca**^2 + ^dynamics, the simplicity of the FHN model makes it an attractive candidate for modeling the propagation of depolarization waves in such large 2D and 3D simulations as the numerical examples presented in this work. We propose a general approach to design the conductivity tensor orientation for any uterine shape and estimate the conductivity tensor values in the extracellular and intracellular domains, using Archie’s law [23]. We illustrate our modeling approach using a spherical, four-compartment volume conductor geometry. As we elaborate later on, certain aspects of it stand as a fair approximation of the volume conductor geometry when performing MMG measurements.

The notational convention adopted in this paper is as follows: italic font indicates a scalar quantity, as in *a*; lowercase boldface indicates a vector quantity, as in ***a***, except for vector fields used in Maxwell’s equations such as electric field ***E***, magnetic field ***B***, and current density ***J***; upper case italic indicates a matrix quantity, as in *A*. The matrix transpose is indicated by a superscript “^*T*^” as in *A*^*T*^, and the identity matrix of size *n*×*n* is denoted *I*_*n*_. The set $S^{n}$ denotes the vector space of symmetric *n*×*n*matrices, and the spaces of nonnegative definite matrices and positive definite matrices are denoted by $S_{+}^{n}$ and $S_{++}^{n}$, respectively. The inner product and norm defined in the Euclidean space are denoted by 〈·,·〉 and ∥·∥, respectively.

## Methods

In this section we discuss the electromagnetic source representation of uterine contractions. We will introduce a four-compartment volume conductor model formed by an anisotropic bidomain myometrium and will present the models for the extrauterine electrical potential, magnetic field, and myometrial current source density. Finally, we will present a numerical example to illustrate our modeling approach.

### Volume conductor model

**Figure 3:**
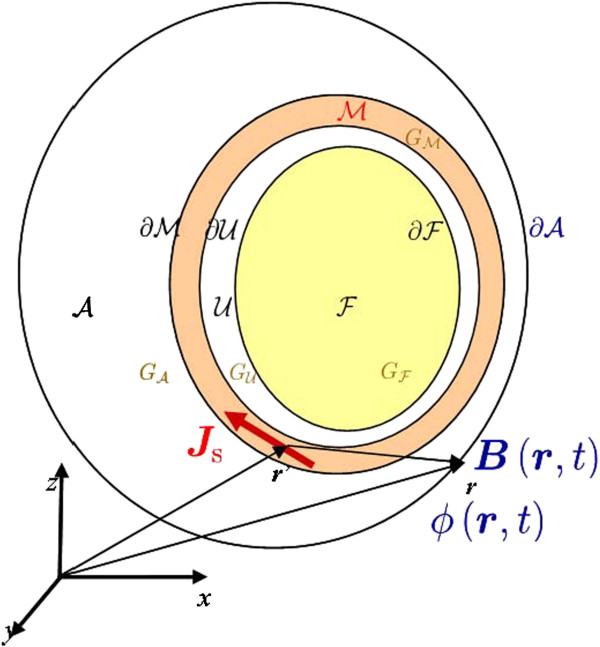
Representation of the four-compartment volume conductor geometry and the forward electromagnetic problem of uterine contractions.

Figure 3 illustrates the four-compartment volume conductor geometry for our problem, where A represents the abdominal cavity and ∂A the boundary surface defined by the abdomen, ℳ represents the myometrium, and ∂ℳ and ∂U are its external and internal boundary surfaces, respectively. The volume denoted by U represents the space filled with the amniotic fluid that exists between the internal uterine wall ∂U and the boundary ∂F defined by the fetus volume F. The vectors ***r***and ***r***^′^indicate the positions of the observation point and source, respectively, with respect to the main axis of reference.

### Extrauterine magnetic field and electrical potential

The electromagnetic properties of uterine contractions can be analized by solving a set of Maxwell’s equations [24,25] subject to boundary conditions given by the volume conductor geometry. In general, in a passive non-magnetic medium the total current density is the sum of the ohmic volume current and the polarization current, also known as the displacement current [24,25]. The ohmic current is the result of ions flowing in the medium and the displacement current is the result of a time varying electric field [24,25]. If the temporal variations of the electric field and the magnetic field, i.e. low frequencies, are small enough that the displacement current is very small with respect to the ohmic current, then it is valid to model the electromagnetic phenomena using the quasi-static approximation of the Maxwell Equations. Under the quasi-static approximation, changes in the sources that generate the electromagnetic field affect all field quantities instantaneously in the whole domain, and the total current density in the volume conductor geometry will be the sum of ohmic currents only. Biolectromagnetic fields vary slowly in time, with frequency components below 1KHz and with a spatial characteristic length scale several times much larger than the volume conductor of interest [26], i.e, much larger than the diameter of the uterus. Hence, changes in the bioelectric sources affect the bioelectromagnetic field in the volume conductor geometry instantaneously, which justifies the use of the quasi-static approximation of Maxwell’s equations [25,26]. Therefore, the extrauterine magnetic field ***B***(***r***,*t*) at a position ***r***and instant *t* is given as follows:

(1) $$\nabla \times \frac{1}{\mu_{o}}B(r,t)=J(r,t),$$

where *μ*_o_ is the permeability of free space and ***J***(***r***,*t*) is the total current density (in A/m^2^). ***J***(***r***,*t*)is given by

(2) $$J\left(r,t\right)=J_{s}\left(r,t\right)+G\left(r\right)E\left(r,t\right),$$

where ***J***_s_(***r***,*t*) is the uterine current density source and *G*(***r***)***E***(***r***,*t*)is the conduction current density (or return currents), as described by Ohm’s law, ***E***(***r***,*t*) denoting the electric field established by ***J***_s_(***r***,*t*)and $G\left(r\right)\in S_{++}^{3}$ denoting the conductivity tensor defined by each compartment. Then, from the quasi-static conditions, ∇·***J***(***r***,*t*)=0, so ∇·*G*(***r***)***E***(***r***,*t*)=−∇·***J***_s_(***r***,*t*). Moreover, since ∇×***E***(***r***,*t*)=0, it follows that ***E***(***r***,*t*)=−∇*ϕ*(***r***,*t*), where *ϕ*(***r***,*t*) denotes the potential. Thus, the equation that governs the relationship between electromyogram potentials and uterine current sources is

(3) $$\nabla \cdot G\left(r\right)\nabla \phi \left(r,t\right)=\nabla \cdot J_{s}\left(r,t\right).$$

Solving the forward electromagnetic problem of uterine contractions requires the computation of ***B***(***r***,*t*) and *ϕ*(***r***,*t*) at ∂A using Eqs.(1) and (3), assuming that ***J***_s_(***r***,*t*)is known in ℳ and *G*(***r***) is known in all domains defined by the volume conductor geometry (see Figure 3).

The biological current sources ***J***_s_(***r***,*t*)in the myometrium are the transmembrane ionic fluxes due to concentration gradients, which flow across the surface membrane of the myocyte (smooth cells) from the extracellular medium into the intracellular medium and vice versa. The density of these ionic currents is also referred to as the impressed current density, since its origin is non electrical in nature, and it is the primary cause for the establishment of an electric field that induces secondary density currents in a conductive domain. We will model ***J***_s_(***r***,*t*) using a bidomain approach, which has proved to be a successful method to study electrophysiological activity in the myocardium [21,22] and, more recently, in the uterus [27].

### Myometrial current-source density model

**Figure 4:**
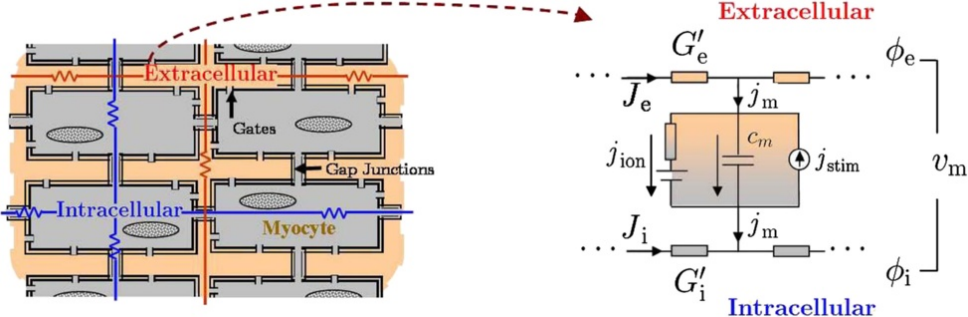
Illustration of the bidomain modeling approach.

In the myometrium, the intracellular and extracellular domains are both physically connected through membrane gates, and the intracellular domain is connected though gap junctions [1,19]. Therefore, we model the myometrium using the bidomain modeling approach. This approach represents the tissue (myometrium) as two interpenetrating extra-intracellular continuous domains with different conductivity values along and across the direction of the fiber [21,22], and it models the tissue using the generalized-passive cable equation. The bidomain modeling approach was originally derived for modeling the propagation of the transmembrane potential of the myocardium and proved to be a successful approach to study the functions of the heart [21,22]. Figure 4 shows a simplified illustration of the tissue and the bidomain approach, where *ϕ*_i_(***r***,*t*) and *ϕ*_e_(***r***,*t*) are the intracellular and interstitial potentials, respectively, and *v*_m_(***r***,*t*)=*ϕ*_i_(***r***,*t*)−*ϕ*_e_(***r***,*t*)is transmembrane potential. The conductivity tensors in the intracellular and extracellular domains are denoted by $G_{i}^{\prime }$ and $G_{e}^{\prime }$ (in S/m), and, using Ohm’s law, the current densities in each domain are given by $J_{i,e}\left(r,t\right)=-G_{i,e}^{\prime }\nabla \phi_{i,e}\left(r,t\right)$. The transmembrane volume current density in (A/m^3^) is denoted by *j*_m_(***r***,*t*) and is given by

(4) $$j_{m}\left(r,t\right)=a_{m}\;c_{m}\frac{\partial v_{m}}{\mathrm{\partial t}}+j_{\text{ion}}-j_{\mathrm{stim}},$$

(5) $$\begin{array}{l} \;=a_{m}\;\left(c_{m}\frac{\partial v_{m}}{\mathrm{\partial t}}+J_{\text{ion}}-J_{\text{stim}}\right), \end{array}$$

where *j*_ion_(***r***,*t*) is the ionic volume current density (in A/m^3^) of a myocyte, *j*_stim_(***r***,*t*) is the stimulus volume current density (in A/m^3^), *c*_m_ is the membrane capacitance per unit area (in F/m^2^), and *a*_m_ is the surface-to-volume ratio of the membrane (in 1/m). To summarize the above description, the bidomain approach models the signal propagation in the tissue using the generalized passive cable equation model (Figure 4) which considers the current densities flowing through the extracellular domain given by ***J***_e_(***r***,*t*), flowing through the intracellular domain given by ***J***_i_(***r***,*t*), and connecting both media given by *j*_m_(***r***,*t*).

Applying the conservation of charge to both domains, we obtain the following relationships:

(6) $$\nabla \cdot J_{e}\left(r,t\right)=j_{m}\left(r,t\right),\text{and}$$

(7) $$\nabla \cdot J_{i}\left(r,t\right)=-j_{m}\left(r,t\right).$$

Adding (6) and (7), we have ∇·(***J***_i_(***r***,*t*) + ***J***_e_(***r***,*t*))=0. Hence, the total current density in the myometrium is given by

(8) $$J\left(r,t\right)=-G_{i}^{\prime }\nabla \phi_{i}\left(r,t\right)-G_{e}^{\prime }\nabla \phi_{e}\left(r,t\right),\;r\in ℳ,$$

which can be expressed in terms of *v*_m_(***r***,*t*)and *ϕ*_e_(***r***,*t*) as follows:

(9) $$J\left(r,t\right)=-G_{i}^{\prime }\nabla v_{m}\left(r,t\right)-G_{ℳ}^{\prime }\nabla \phi_{e}\left(r,t\right),\;r\in ℳ,$$

where $G_{ℳ}^{\prime }=\left(G_{e}^{\prime }+G_{i}^{\prime }\right)\in S_{++}^{3}$ is the bulk myometrium conductivity tensor. Since spatial variations of *v*_m_(***r***,*t*)depend on the local establishment of a transmembrane current density, *j*_m_(***r***,*t*)≠0, we define the impressed current-density source as $J_{s}(r,t)=-G_{i}^{\prime }\nabla v_{m}(r,t)$. Note that ***J***_s_(***r***,*t*)exists only when the spatial gradient exists, i.e., only in a region where the myometrium is undergoing depolarization (excitation) or repolarization.

The total current at the myometrium ***J***(***r***,*t*)depends on the spatio-temporal variations of *v*_m_(***r***,*t*)and *ϕ*_e_(***r***,*t*), which are governed by the system of equations formed by Eqs. (5), (6) and (7). Using simple algebraic manipulations, the aforementioned system of equations can be written in terms of *v*_m_(***r***,*t*) and *ϕ*_e_(***r***,*t*) only, obtaining the following equivalent expressions:

(10) $$\begin{array}{lll} \;\nabla \cdot G_{i}^{\prime }\nabla (v_{m}\left(r,t\right)+\phi_{e}\left(r,t\right)) & =a_{m}\;\left(c_{m}\frac{\partial v_{m}\left(r,t\right)}{\mathrm{\partial t}}\right)\; & \;\\ & \;\left(+\;J_{\text{ion}}\;\left(r,t\right)\;-\;J_{\text{stim}}\;\left(r,t\right)\;\;\right)\;,\; \end{array}$$

(11) $$\nabla \cdot (G_{i}^{\prime }+G_{e}^{\prime })\nabla \phi_{e}\left(r,t\right)=-\nabla \cdot G_{i}^{\prime }\nabla v_{m}\left(r,t\right).$$

This set of reaction-diffusion equations is also known as the bidomain equations [21,22]. The diffusion part of the equations governs the spatial evolution of both the transmembrane and extracellular potentials, and the reaction part is given by the local ionic current cell dynamics. The solutions for *v*_m_(***r***,*t*) and *ϕ*_e_(***r***,*t*) depend on ***J***_ion_(***r***,*t*), ***J***_stim_(***r***,*t*), and the conductivity tensors, in addition to the boundary and the initial conditions. Since our goal is to model the propagation of electrical activity in the myometrium, we are interested in the class of traveling-wave solutions of these equations whose waveform depends on ***J***_ion_(***r***,*t*) and whose initiation depends on ***J***_stim_(***r***,*t*). In what follows, we describe the models for both current densities, ***J***_ion_(***r***,*t*)and ***J***_stim_(***r***,*t*), and for the conductivity tensors, $G_{i}^{\prime }$ and $G_{e}^{\prime }$.

#### Ionic current model

The predominant type of transmembrane-potential waveforms measured in the pregnant human myometrium are spikes and plateau [1,28-31]. In this work, we focus on modeling the plateau-type transmembrane potential, as it has been more frequently observed [1,28-30]. In particular, we model ***J***_ion_(***r***,*t*), using a variation of the FitzHugh-Nagumo (FHN) equations [32-34], as follows:

(12) $$\begin{array}{l} J_{\text{ion}}(r,t)=-\frac{1}{\varepsilon_{1}}\left(k\;\left(v_{m}-v_{1}\right)\left(v_{2}-v_{m}\right)\right)\\ \;\left(\;\;\times \left(v_{m}-v_{3}\right)-w\right),\text{and}\; \end{array}$$

(13) $$\begin{array}{l} \;\frac{\mathrm{\partial w}}{\mathrm{\partial t}}=\varepsilon_{2}\left(\beta v_{m}-\mathrm{\gamma w}+\delta \right), \end{array}$$

where *ε*_1_, *ε*_2_, *k*, *v*_1_, *v*_2_, *v*_3_, *δ*, *γ*, and *β*are model constants, and *w* (in V) is a state variable of the model. The parameter *ε*_1_ (in *Ω*m^2^) controls the sharpness of the leading and trailing edges of the action potential waveform; the smaller *ε*_1_ is, the more vertical the edge is. Note that *ε*_1_is a quantity of resistivity, therefore the smaller its value, the greater the permeability of the membrane to ionic flux. The parameter *ε*_2_ (in s^−1^) controls the action potential duration; the smaller *ε*_2_ is, the longer it takes a cell to recover. The parameters *v*_1_, *v*_2_, *v*_3_ (in V), and *k* (in 1/V^2^) control the range of *v*_m_(***r***,*t*). Note that for a given set of *k*,*v*_1_, *v*_2_, and *v*_3_, the ratios *β*/*γ* and *δ*/*γ* control the excitability threshold of the cell. The larger *β*/*γ*is, the lower the excitability threshold that sets the cell dynamic to an oscillatory stable behavior between resting and exciting states is. Over a certain value, the cell dynamic becomes bistable, that is, if the cell starts from a resting potential, it changes to an excited state and remains there. On the other hand, a very negative *β*/*γ* value results in a permanent resting state. In the Results Section, presented further below, we select the model parameters using phase-space analysis and using the transmembrane potentials recorded from isolated human myometrial strips at term as a reference [13,30]. This model does not consider explicitly the **Ca**^2 + ^dynamics, and, moreover, it assumes that changes in the intra- and extra-cellular ion concentrations are insignificant even after several depolarizations. However, its simplicity facilitates the modeling of the propagation of depolarization waves in large 2D and 3D domains.

#### Stimulus current model

We also introduce a temporal-spatial model for ***J***_stim_, representing the stimulus due to pacemaker areas [1,19], as follows:

(14) $$J_{\text{stim}}(r,t)=\frac{1}{\varepsilon_{1}}\overset{N_{p}}{\underset{i=1}{\sum }}\nu_{i}h_{i}(r,t),$$

where *h*_*i*_(*r*,*t*) is a spatio-temporal function with range in [0,1], *ν*_*i*_ is the amplitude (in V), and *N*_p_is the number of pacemaker areas. Intuitively, the former should modify the excitability of the cell at a certain instant of time based on the threshold value. In particular, our model assumes that the uterine myocyte can act as either a pacemaker or pace-follower, specifically, the spontaneous electrical behavior exhibited by the myometrium is an inherent property of the uterine myocyte (see [19] for more details.) Note that the size, duration, and intensity of the pacemaker area need to be chosen such that a stable traveling waveform solution to the bidomain equations on the myometrium is possible.

#### Conductivity tensor model

The structure of the fasiculata within the uterus has not been completely characterized as a whole. Despite of not having a sense of a global uterine-fiber arcuitecture, it has been possible to characterize local structures in each of the three layers of the uterine wall [5,6,12]. In [5], the authors investigated the global fiber architecture of the non-pregnant uterus by diffusion tensor magnetic resonance imaging (DTMRI). From the ex-vivo analysis of five non-pregnant uteri, the authors identified an inner circular layer around the uterine cavity on slices orthogonal to the long axis of the organ. In the regions outside the inner circular layer, they could not identify a global structure but did find several locally aligned groups of fibers. At the level of the cervix, they found an outer circular layer and an inner region with mostly longitudinal components. In [12], from the analysis of the DTMRI of one postpartum uterus, the authors concluded that the myometrium is organized into bundles, with the fibre bundles forming interweaving sheets. In the following, we will introduce an approach for designing the conductivity tensors in the myometrium.

Assume that the conductivity tensors are diagonal in a local coordinate system that is defined with respect to each myocyte and characterized by the unit vectors {***e***_1_,***e***_2_,***e***_3_}. In particular, *G*_i_ and *G*_e_ are diagonal matrices $\in S_{++}^{3}$ given by

(15) $$\begin{array}{ll} G_{i}=\left(\begin{array}{ccc} \sigma_{\text{ix}} & 0 & 0\\ 0 & \sigma_{\text{iy}} & 0\\ 0 & 0 & \sigma_{\text{iz}} \end{array}\right), & G_{e}=\left(\begin{array}{ccc} \sigma_{\text{ex}} & 0 & 0\\ 0 & \sigma_{\text{ey}} & 0\\ 0 & 0 & \sigma_{\text{ez}} \end{array}\right) \end{array}.$$

In order to take into account variable fiber orientation in the myometrium, we need to describe it in a global Cartesian coordinate system in which the local basis is defined at any point ***r*** as *A***=**[***a***_1_(***r***) ***a***_2_(***r***) ***a***_3_(***r***)], where ***a***_3_(***r***)is parallel to the main fiber axis. The representation of the tensors *G*_i_and *G*_e_ in terms of a global coordinate system is given by

(16) $$G_{i}^{\prime }=AG_{i}A^{T},\;\;G_{e}^{\prime }=AG_{e}A^{T}.$$

Assuming that the myocyte fiber conductivities in both domains are cylindrically symmetric, then *σ*_ex_=*σ*_ey_=*σ*_et_, *σ*_ix_=*σ*_iy_=*σ*_it_, *σ*_iz_=*σ*_il_, and *σ*_ez_=*σ*_el_. Therefore, the conductivity tensors can be expressed as follows [35]:

(17) $$G_{i}^{\prime }=\left(\sigma_{\text{il}}-\sigma_{\text{it}}\right)a_{3}(r)a_{3}^{T}(r)+\sigma_{\text{it}}\;I_{3},\text{and}$$

(18) $$G_{e}^{\prime }=\left(\sigma_{\text{el}}-\sigma_{\text{et}}\right)a_{3}(r)a_{3}^{T}(r)+\sigma_{\text{et}}\;I_{3}.$$

Hence, to construct the conductivity tensors as a function of ***r***, it is enough to define the vector field ***a***_3_(***r***), the myometrial fiber orientation, in each location of the anisotropic domain, as well as the conductivity values *σ*_il_, *σ*_el_,*σ*_it_,and *σ*_et_, because of the assumption of cylindric symmetry.

##### Designing myometrial fiber orientations

To design ***a***_3_(***r***) at each point ***r***, we represent the uterus as a hollow volume with uniform thickness, and we describe it as a union of mutually disjoint closed surfaces or layers. We use the implicit definition of a surface, namely, the set of points ***r*** satisfying *f*(***r***)=0. Then, at each point ***r***, we define a set of local orthonormal coordinate axes given by $\left\{\hat{n}(r),{\hat{t}}_{1}(r\right)$, ${\hat{t}}_{2}(r)\}$, where $\hat{n}(r)=\frac{\nabla f(r)}{∥\nabla f(r)∥}$ is the normal vector to the layer containing ***r***, and ${\hat{t}}_{1}(r)$ and ${\hat{t}}_{2}(r)$ are mutually orthogonal vectors that belong to the tangent plane of the respective layer at point ***r***. We define ${\hat{t}}_{1}(r)$ and ${\hat{t}}_{2}(r),$ using the curve of symmetry of the uterine inner-circular layer as a reference [5]. This curve goes from the fundus to the cervix, and it coincides with the long axis of the non-pregnant uterus. We denote *C* as the curve of symmetry using the following parametric representation as a function of a single parameter *t*:

(19) $$C:t\mapsto r_{C}\left(t\right),\;t_{1}\leq t\leq t_{2},$$

where ***r***_*C*_(*t*)is a point defined with respect to the global coordinate system, and ***r***_*C*_(*t*_1_) and ***r***_*C*_(*t*_2_) are the extreme points of the curve. For example, let ***r***_*C*_(*t*)=(*x*(*t*),*y*(*t*),*z*(*t*)) with respect to the Cartesian system. Define $\hat{k}(r)=({\left(\frac{dr_{C}\left(t\right)}{\text{dt}}\right|}_{t_{0}}/∥{\left(\frac{dr_{C}\left(t\right)}{\text{dt}}\right|}_{t_{0}}∥$, the unitary vector field with direction given by the tangent vector of ***r***_*C*_(*t*) at *t*_0_, where ***r***_*C*_(*t*_0_) is the closest point to *r* such that $\langle {\left(\frac{dr_{C}\left(t\right)}{\text{dt}}\right|}_{t_{0}},\overset{âƒ—}{r_{C}\left(t_{0}\right)\;r}\rangle =0$. Then, we define ${\hat{t}}_{1}(r)$ to be contained in the plane formed by $\hat{k}(r)$ and $\hat{n}(r)$ as follows:

(20) $${\hat{t}}_{1}(r)=\beta \hat{k}(r)+\gamma \hat{n}(r),$$

subject to the following conditions:

(21) $$\langle {\hat{t}}_{1}(r),\hat{n}(r)\rangle =0,\text{and}$$

(22) $${\langle {\hat{t}}_{1}(r),{\hat{t}}_{1}(r)\rangle }^{2}=1.$$

Therefore, replacing (20) in (21) and (22) we obtain the following system of equations:

(23) $$\begin{array}{l} \;\beta \langle \hat{k}(r),\hat{n}(r)\rangle +\gamma =0,\text{and} \end{array}$$

(24) $$\beta^{2}+\gamma^{2}+2\;\beta \;\gamma \langle \hat{k}(r),\hat{n}(r)\rangle =1.$$

**Figure 5:**
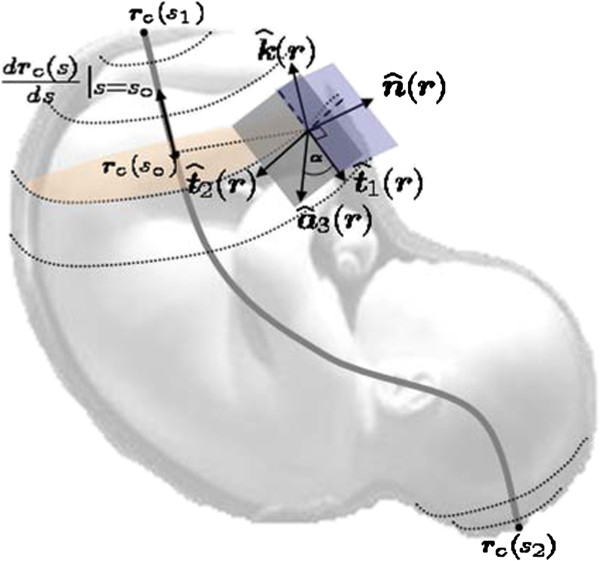
**Simplified illustrations of*a***_**3**_**(*r*)with respect to the local coordinates axis given by**$\{\hat{n}\left(r\right),{\hat{t}}_{1}\left(r\right)$**,**${\hat{t}}_{2}\left(r\right)\}$**.** The blue plane contains the vectors $\hat{n}(r)$, $\hat{k}(r)$, and ${\hat{t}}_{1}(r)$, and it is perpendicular to the gray plane formed by vectors ${\hat{t}}_{1}(r)$, ${\hat{t}}_{1}(r)$ and ***a***_3_(***r***). The orange plane is the cross section of the uterus perpendicular to the vector ${\left(\frac{dr_{C}\left(t\right)}{\text{dt}}\right|}_{t_{0}}$. The gray curve is the curve of symmetry ***r***_*C*_(*t*)with ***r***_*C*_(*t*_1_)and ***r***_*C*_(*t*_2_)extreme points of the curve.

Solving for all ***r***, such that $\langle \hat{k}(r),\hat{n}(r)\rangle \neq 1$, we obtain $\beta =\pm \frac{1}{\sqrt{1-\langle \hat{k}(r),\hat{n}(r)\rangle }}$ and $\gamma =\mp \frac{\langle \hat{k}(r),\hat{n}(r)\rangle }{\sqrt{1-\langle \hat{k}(r),\hat{n}(r)\rangle }}.$ Then ${\hat{t}}_{2}(r)={\hat{t}}_{1}(r)\times \hat{n}(r)=\beta \hat{k}(r)\times \hat{n}(r)$, since by definition it is mutually orthogonal to ${\hat{t}}_{1}(r)$. Hence, given ${\hat{t}}_{1}(r)$ and ${\hat{t}}_{2}(r),$ we define ***a***_3_(***r***) as follows:

(25) $$a_{3}(r)={\hat{t}}_{1}(r)\cos\left(\alpha \right)+{\hat{t}}_{2}(r)\sin\left(\alpha \right),$$

where *α* is the fiber orientation angle with respect to ${\hat{t}}_{1}(r)$. In order to take into account complex fiber orientations *α*can be modeled as a spatial function defined over the domain of interest. Given our uterine volume assumptions, the points ***r***_*C*_(*t*_1_) and ***r***_*C*_(*t*_2_) are the only points that satisfy the condition $\langle \hat{k}(r),\hat{n}(r)\rangle =1$. Since at ***r***_*C*_(*t*_1_) and ***r***_*C*_(*t*_2_) we cannot define ${\hat{t}}_{1}(r)$ and ${\hat{t}}_{2}(r)$ using the curve *C* as a global reference, we set ***a***_3_(***r***)=0, defining a point of isotropic conductivity. In Figure 5 we represent the fiber orientation ***a***_3_(***r***) with respect to the local coordinate axes given by $\left\{\hat{n}(r),{\hat{t}}_{1}(r),{\hat{t}}_{2}(r)\right\}$.

If we have a uterine volume such that *C* is parallel to the *z* axis, then ***a***_3_(***r***) can be written as a function of $\hat{n}(r)$ as follows:

(26) $$a_{3}(r)=\left(P\cos\left(\alpha \right)+F\sin\left(\alpha \right)\right)\hat{n}(r),$$

where

(27) $$\begin{array}{lll} P & =\left[\begin{array}{lll} a & 0 & 0\\ 0 & a & 0\\ 0 & 0 & -1/a \end{array}\right],F=\left[\begin{array}{lll} 0 & b & 0\\ -b & 0 & 0\\ 0 & 0 & 0 \end{array}\right],\;\\ a & =\frac{\nabla_{z}f(r)}{\sqrt{{\left(\nabla_{x}f(r)\right)}^{2}+{\left(\nabla_{y}f(r)\right)}^{2}}},\text{and}\; & \; \end{array}$$

(28) $$b=\frac{∥\nabla f(r)∥}{\sqrt{{\left(\nabla_{x}f(r)\right)}^{2}+{\left(\nabla_{y}f(r)\right)}^{2}}},$$

with ∇_*j*_the *j*-th component of the gradient. In the case of a spherical myometrium, ***a***_3_(***r***) is given as by

(29) $$a_{3}(r)=\left[\begin{array}{l} \frac{\text{zx}\cos\alpha }{\sqrt{x^{2}+y^{2}}R}+\frac{y\sin\alpha }{2\sqrt{x^{2}+y^{2}}}\\ \frac{\text{zy}\cos\alpha }{\sqrt{x^{2}+y^{2}}R}-\frac{x\sin\alpha }{\sqrt{x^{2}+y^{2}}}\\ -\frac{\sqrt{x^{2}+y^{2}}}{R}\cos\alpha \end{array}\right],$$

where $R=\sqrt{x^{2}+y^{2}+z^{2}.}$ Note that, for *α*=0, the main axis of the fibers runs vertically from the fundus to the cervix.

##### Estimating myocyte fiber conductivities

To the best of our knowledge, values of the intracellular and extracellular conductivity tensors have not been reported for the human myocyte, and therefore, these have to be estimated. To model the extracellular conductivity values *σ*_el_ and *σ*_et_, we assume a grid-type distribution of myocytes in the myometrium and use an estimate of the extracellular conductivity the human myometrium obtained by applying Archie’s law [23]. We describe myocytes as long cylinders with diameter *d*_cell_ and axis length *l*_cell_, such that *d*_cell_≪*l*_cell_Assuming that myocytes are uniformly arranged in a cubical grid with length *l*_T_=*l*_cell_ + 2*Δ*_e_ and whose cross section has sides *d*_T_=*d*_cell_ + 2*Δ*_e_,then we have *σ*_el_and *σ*_et_ as follows:

(30) $$\sigma_{\text{el}}={\tilde{\sigma }}_{e}\left(1-\frac{\Pi {\left(\frac{d_{\text{cell}}}{2}\right)}^{2}}{d_{T}^{2}}\right)\text{and}$$

(31) $$\sigma_{\text{et}}={\tilde{\sigma }}_{e}\left(1-\frac{d_{\text{cell}}l_{\text{cell}}}{d_{T}l_{T}}\right),$$

where ${\tilde{\sigma }}_{e}$ is the conductivity of the extracellular medium in the myometrium. ${\tilde{\sigma }}_{e}$ can be computed using the effective myometrium conductivity $\sigma_{ℳ}$, available in the literature, and Archie’s law [23] as follows:

(32) $${\tilde{\sigma }}_{e}=\frac{\sigma_{ℳ}}{{\left(1-p\right)}^{m}},$$

where *p* is the fraction of the volume occupied by the myocytes and collagenous fibers in the tissue, and *m* is the so-called cementation factor, which depends on the shape and orientation of the myocyte in the tissue.

To compute the intracellular conductivities *σ*_il_and *σ*_it_, we assume that the intracellular and extracellular domains have equal anisotropy ratios, that is,

(33) $$G_{i}^{\prime }=\varsigma G_{e}^{{}^{\prime }},$$

and thus we need to compute *ς*. We obtain an analytical expression for *ς*using reported values of the propagation speed of a transmembrane potential waveform traveling on isolated tissue strips from pregnant human myometrium at term [28]. In particular, replacing (33), (12), and (13) in the bidomain equations (10) and (11), and solving *v*_m_(***r***,*t*) for a traveling wave solution *v*_m_(***ξ·r−****ct*), where ***ξ*** is a unitary vector pointing along the main axis of the myocyte and *c* is the speed of propagation, we obtain the following expression for *ς*:

(34) $$\varsigma =g\left(\frac{2\;c^{2}\varepsilon_{1}a_{m}\;c_{m}^{2}}{\sigma_{\text{el}}\;k\;{\left(v_{1}^{*}-2\;v_{2}^{*}+v_{3}^{*}\right)}^{2}}\right),$$

where $g\left(x\right)=\frac{x}{1-x}$. Further, $v_{1}^{*}$, $v_{2}^{*}$, and $v_{3}^{*}$ are the roots of the following polynomial in *v*_*m*_:

(35) $$f\left(v_{m}\right)=\left(v_{m}-v_{1}\right)\left(v_{2}-v_{m}\right)\left(v_{m}-v_{3}\right)-\frac{1}{\mathrm{k\gamma}}\left(\beta v_{\text{mr}}+\delta \right),$$

with *v*_mr_denoting the resting transmembrane potential of the human myocyte. Note that in order to have *ς*≥0, *ε*_1_ must satisfy the following inequality:

(36) $$0<\varepsilon_{1}<\frac{\sigma_{\text{el}}\;k\;{\left(v_{1}^{*}-2\;v_{2}^{*}+v_{3}^{*}\right)}^{2}}{2\;c^{2}a_{m}\;c_{m}^{2}}.$$

### Monodomain approximation and boundary conditions

Assuming an equal anisotropy ratio, Eq. (33), simplifies the solution of the bidomain equations (10) and (11) by decoupling them as follows:

(37) $$\begin{array}{lll} \nabla \cdot \frac{\varsigma }{\left(\varsigma +1\right)}G_{e}^{\prime }\nabla v_{m}(r,t) & =a_{m}\;\left(\;c_{m}\frac{\partial v_{m}(r,t)}{\mathrm{\partial t}}\;+\;J_{\text{ion}}(r,t)\right)\; & \;\\ & \;\left(-J_{\text{stim}}(r,t)\right)\text{in}\;ℳ,\; \end{array}$$

(38) $$\nabla \cdot \frac{\left(\varsigma +1\right)}{\varsigma }G_{e}^{\prime }\nabla \phi_{e}(r,t)=-\nabla \cdot G_{e}^{\prime }\nabla v_{m}(r,t),\;\text{in}\;\mathrm{ℳ.}$$

The above simplification is also known as the monodomain approximation of the bidomain equations, which, under suitable boundary conditions, allows the computation of *v*_m_ and, thus, ***J***_s_, independent from *ϕ*_e_.

To set up boundary conditions for computing electrical potentials, we need to take into account the volume conductor geometry (see Figure 3). In particular, we have two bidomain-monodomain interfaces: one between the myometrium ℳ and abdominal volume A and one between the myometrium and the intrauterine cavity U. Therefore, we have the following boundary conditions: (i) continuity of the interstitial potential *ϕ*_e_ at the perimetrium surface ∂ℳ to the abdomen potential $\phi_{A}$, (ii) flow of the normal component of ***J***that crosses over from the uterus to the abdominal medium, (iii) no flow of the normal component of ***J***_s_to the abdominal medium, (iv) continuity of the interstitial potential *ϕ*_e_ at the endometrium surface ∂U to the intrauterine cavity potential $\phi_{U}$, (v) flow of the normal component of ***J***that crosses over from the uterus to the intrauterine cavity filled with amniotic fluid, (vi) no flow of the normal component of ***J***_s_ to the intrauterine cavity, (vii) no flow of the normal component of ***J***that crosses over from the abdominal cavity to air, and (viii) either no flow or else flow of the normal component of ***J*** that crosses over from the intrauterine cavity, filled with amniotic fluid, to the fetus, depending on if it is covered with vernix caseosa (*λ*=0) or not (*λ*≠0) [36]. These boundary conditions are summarized as follows:

(39) $$\begin{array}{l} \;\phi_{e}(r,t)=\phi_{A}(r,t),\;\text{in}\;\partial ℳ, \end{array}$$

(40) $$\begin{array}{l} \;{\hat{n}}_{ℳ}\cdot (G_{i}^{\prime }\nabla \phi_{i}(r,t)+G_{e}^{\prime }\nabla \phi_{e}(r,t))={\hat{n}}_{ℳ}\cdot G_{A}\nabla \phi_{A}(r,t),\\ \;\;\text{in}\;\partial ℳ,\; \end{array}$$

(41) $$\begin{array}{l} \;{\hat{n}}_{ℳ}\cdot G_{i}^{\prime }\nabla v_{m}(r,t)=0,\;\text{in}\;\partial ℳ, \end{array}$$

(42) $$\begin{array}{l} \;\phi_{e}(r,t)=\phi_{U}(r,t),\;\text{in}\;\partial U \end{array}$$

(43) $$\begin{array}{l} {\hat{n}}_{U}\cdot (G_{i}^{\prime }\nabla \phi_{i}(r,t)+G_{e}^{\prime }\nabla \phi_{e}(r,t))={\hat{n}}_{U}\cdot G_{U}\nabla \phi_{U}(r,t),\\ \;\;\;\text{in}\;\partial U,\; \end{array}$$

(44) $$\begin{array}{l} \;{\hat{n}}_{U}\cdot G_{i}^{\prime }\nabla v_{m}(r,t)=0,\;\text{in}\;\partial U, \end{array}$$

(45) $$\begin{array}{l} \;{\hat{n}}_{A}\cdot G_{A}\nabla \phi_{A}(r,t)=0,\;\text{in}\;\partial A \end{array}$$

(46) $$\begin{array}{l} \;{\hat{n}}_{F}\cdot G_{U}\nabla \phi_{U}(r,t)=\lambda \left({\hat{n}}_{F}\cdot G_{F}\nabla \phi_{F}(r,t)\right)\\ \;\;\text{in}\;\partial F,\; \end{array}$$

where ${\hat{n}}_{j}$ is the normal vector to the surface *j* in each case.

### Summary of modeling assumptions

In this section we list all the assumptions that were considered while developing our model: 

•**Volume conductor geometry:** it is given by four spherical compartments. The uterine wall is a single layerwith uniform thickness. The abdominal, intrauterine, and fetal compartments have isotropic and homogeneous conductivity. The uterine compartment is anisotropic and homogeneous. All boundaries between compartments, except for the external boundary of the abdominal and fetal compartments, are electrically conductive. The fetal compartment boundary can be set to be electrically insulated or electrically conductive to take into account the presence of vernix caseosa.

•**Myometrium:** It is a continuous medium formed by array of myocytes with same transmembrane potential shape across the whole domain. The extracellular and intracellular tissue conductivities are anisotropic, homogeneous, and proportional to each other(equal anisotropic ratio). The fiber orientation with respect to the main symmetry axis is kept fix across the whole domain.

•**Myocyte:** It is a cell of cylindrical shape. Its electrical conductivity is assumed to have a cylindrical symmetry with higher conductivity along the main axis of the cylinder than along the axis defining the cross section. Transmembrane potential shape is plateau-type, and any myocyte can play the roll of pacemaker or follower by means of a stimulus current.

### Numerical computation of *v*_m_(***r***,*t*), *ϕ*(***r***,*t*), and ***B***(***r***,*t*)

The computations of *v*_m_(***r***,*t*), *ϕ*(***r***,*t*), and ***B***(***r***,*t*)are given by the following procedure: 

•**Step 1**: Solve for *v*_m_(*r*,*t*)using Eqs. (37), (12), (13), and (14) subject to boundary conditions (41) and (44), and to initial conditions given by

(47) $$\begin{array}{l} \;v_{m}(r,0)=v_{\text{mr}}, \end{array}$$

(48) $$\begin{array}{l} \;w_{m}(r,0)=\left(\frac{\beta }{\gamma }v_{\text{mr}}+\frac{\delta }{\gamma }\right), \end{array}$$

(49) $$\frac{\partial v_{m}(r,0)}{\mathrm{\partial t}}=0,\text{and}$$

(50) $$\frac{\partial w_{m}(r,0)}{\mathrm{\partial t}}=0.$$

•**Step 2**: Solve for *ϕ*_e_(*r*,*t*)in ℳ and *ϕ*(*r*,*t*) in A and U, using the solution of *v*_m_(*r*,*t*), computed in Step 1, and the following expressions:

(51) $$\nabla \cdot \frac{\left(\varsigma +1\right)}{\varsigma }G_{e}^{\prime }\nabla \phi_{e}(r,t)=-\nabla \cdot G_{e}^{\prime }\nabla v_{m}(r,t),\;\text{in}\;ℳ,$$

(52) $$\begin{array}{l} \;\nabla \cdot G_{A}\nabla \phi (r,t)=0,\;\text{in}\;A, \end{array}$$

(53) $$\begin{array}{l} \;\nabla \cdot G_{U}^{\prime }\nabla \phi (r,t)=0,\;\text{in}\;U, \end{array}$$

subject to the boundary conditions (39), (40), (42), (43), (45), and (46).

•**Step 3**: Solve for ***B***(***r***,*t*)using Eq.(1), and computing the total current density ***J***(***r***,*t*) in the whole uterine domain, using the solutions of *v*_m_(*r*,*t*), *ϕ*_e_(*r*,*t*), and *ϕ*(*r*,*t*), obtained in Steps 1 and 2, and considering the following boundary conditions:

(54) $$\begin{array}{l} \;{\hat{n}}_{F}\times \left(B_{F}(r,t)-B_{U}(r,t)\right)=0,\text{in}\;\partial F, \end{array}$$

(55) $${\hat{n}}_{U}\times \left(B_{ℳ}(r,t)-B_{U}(r,t)\right)=0,\text{in}\;\partial U,$$

(56) $${\hat{n}}_{ℳ}\times \left(B_{A}(r,t)-B_{ℳ}(r,t)\right)=0,\text{in}\;\partial ℳ,$$

(57) $$\begin{array}{l} \;{\hat{n}}_{A}\times \left(B_{A}(r,t)-B_{E}(r,t)\right)=0,\text{in}\;\partial A, \end{array}$$

(58) $$\begin{array}{l} \;{\hat{n}}_{A}\times B_{E}(r,t)=0,\text{in}\;\partial E, \end{array}$$

where E is an additional volume that must be incorporated in order to compute the magnetic field at the abdominal surface using FEM. The boundary conditions at the interfaces ∂F,∂U,∂ℳ, and ∂A describe the continuity of the magnetic field between domains, and the boundary condition at ∂E, the external boundary of E, is of electric isolation.

To compute the solution in each of the above steps, we use the FEM solver COMSOL Multiphysics, running on a server with eight 64-bit processors at 2.3 GHz, with 32 GB RAM.

## Results

**Figure 6:**
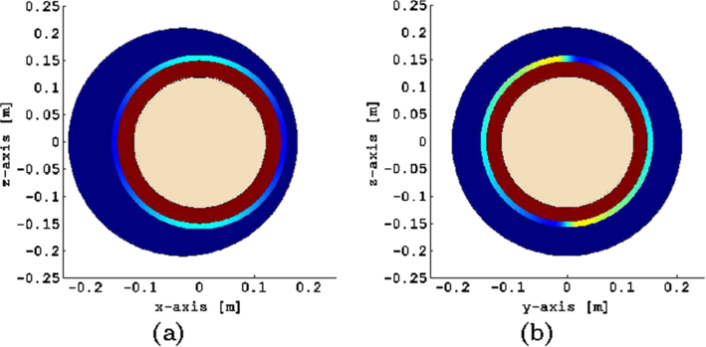
**Four-compartment volume conductor geometry used in the numerical examples.** (**a**) View of z-x plane, and (**b**) z-y plane. Each compartment is assigned a different color. The myometrium has a non-uniform color to denote that its conductivity is anisotropic.

In the following, we illustrate our modeling approach by considering the electrophysiological characteristics of the myometrium and a spherical volume conductor geometry. In Figure 6, we illustrate a spherical, four-compartment volume conductor geometry used in our numerical example. We define a spherical myometrium with a 16 cm radius measured from the center to ∂ℳ and assume the uterine wall has a uniform thickness of 1 cm. We also consider a spherical fetus with a 12 cm radius concentric to the myometrium and fully covered with vernix caseosa, i.e., *λ*=0in (46). The abdominal compartment is also spherical, with a 21 cm radius shifted −3 cm from the center of the myometrium in the x axis. This shift is to take into account that the uterus is closer to the abdominal surface than to the dorsal surface. We set the coordinate axis of reference at the center of the myometrium.

**Figure 7:**
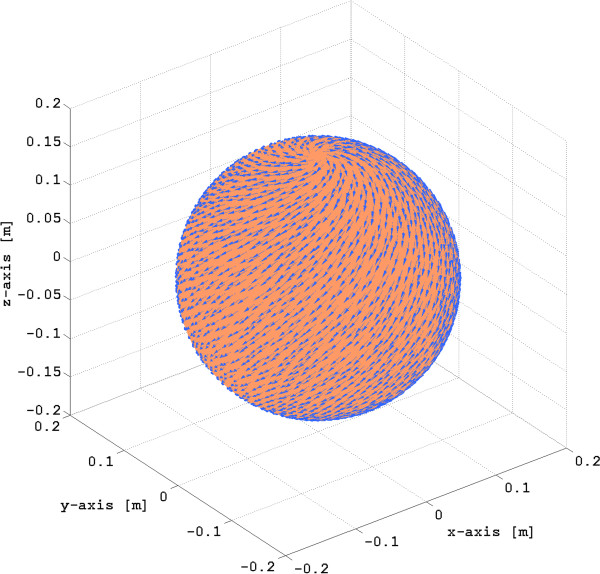
**Geometry and fiber orientation in spherical myometrium given by*α=45*°.**

The conductivity values for each compartment are given in Table 1. In particular, to compute the extracellular myometrial conductivity tensors, we use the average values for the uterine myocyte dimensions at term based on data reported in [1,4,9] (see Table 2). We assume the average human myocyte shape to be a long cylinder with a small cross section; therefore, we use a cementation factor *m*=4/3 (see [23] for more details on the computation of this factor). The volume fraction *p* occupied by myocytes and collagenous fibers in the myometrium is set to 0.6. In order to consider an average myometrial fiber architecture that contains both circularly- and obliquely-oriented fibers we choose the fiber orientation angle *α* to be 45^*o*^. Figure 7 illustrates the global structure of the myometrial fiber orientation for this angle.

**Table 1:** Conductivity values of the volume conductor geometry

**Symbol**	** Value**	**Reference**
$G_{A}$	0.2 S/m	[36]
$G_{U}$	1.74 S/m	[36]
$G_{F}$	0.2 S/m	[36]
*σ*_el_	0.68 S/m	Eq. (30)
*σ*_et_	0.22 S/m	Eq. (31)
*ς*	0.8	Eq. (34)

**Table 2:** Myocyte dimensions and Archie’s law parameters

**Symbol**	**Value**	**Reference**
*d*_cell_	7 *μ*m	[1]
*l*_cell_	450 *μ*m	[1]
*d*_T_	8 *μ*m	
*l*_T_	451 *μ*m	
$\sigma_{ℳ}$	0.5 S/m	[36]
*m*	4/3	[4,23]
*p*	0.6	[5,23]

We select the model parameters of the ionic current model using phase-space analysis, using as a reference the average plateau-type transmembrane potential recorded from isolated tissue strips of human myometrium at term [13,30]. In particular, the average resting potential, considering the results reported from the 37th week of pregnancy onwards, is approximately −56 mV. The plateau has an average depolarization of −27±1mV that terminates in 0.9±0.2minutes by an abrupt repolarization to the resting level [13,30]. Table 3 gives the parameter values used in the numerical example. Note that we compute the surface-area to volume-ratio *a*_m_ using the myocyte dimensions in Table 2.

**Table 3:** Ionic current model parameters

**Symbol**	** Value**	**Reference**
*c*_*m*_	0.01 F/m^2^	[36]
*v*_mr_	−0.056 V	[30]
*a*_*m*_	5.758710^5^ m^−1^	Table 2
*ε*_1_	200 *ω* m^2^	
*ε*_2_	0.09 1/s	
*v*_1_	−0.02 V	
*v*_2_	−0.04 V	
*v*_3_	−0.065 V	
*k*	10^4^ 1/V^2^	
*δ*	0.0520 V	
*γ*	0.1	
*β*	1	
*c*	1.15 cm/sec	[28]

In [19], the authors reported that, in the human uterus, there may be a preferential direction of propagation of contractions, and thus of transmembrane potential propagation, from the fundus toward the isthmus, which could aid in the expulsion of the fetus. Therefore, in order to study this assumption with our model, we consider *j*_stim_ with *N*_*p*_=1, *ν*_1_=2 V, and *h*_1_(*r*,*t*)={1, if 0≤*t*≤0.1, 0.15 ≤∥*r*∥ ≤0.16 and *z*≥0.15 ; 0,otherwise. The size and intensity of the pacemaker area are chosen in order to obtain a stable traveling waveform solution to the bidomain equations on the spherical myometrium.

To solve the set of equations that model the myometrial current-source densities and their electromagnetic fields at the level of the abdomen, we use a three-step procedure along with Finite Element Methods (FEM) (See the Methods Section for more details). The FEM discretization of the whole volume conductor is done using tetrahedral elements. The elements used to discretize each domain consists of 75,964 elements divided as follows: 1,650 elements in the fetus, 7,605 elements in the intrauterine cavity, 8,299 elements in the myometrium, and 19,036elements in the abdominal cavity. To compute the magnetic field using FEM, an additional domain (domain E in (58)) concentric to the volume conductor geometry has to be added. Specifically, we add a concentric sphere with radius of 1m. The discretization of this domain consists of 39,374 elements. The discretized boundaries consists of 10,996triangular elements, and the number of degrees of freedom of the whole domain is 159,130. In all the Steps of the numerical computation procedure described above we use the generalized minimal residual method (GMRES) for solving the resulting system of linear equations after FEM discretization. The backward differentiation formula is used to discretize the time derivative in Step 1.

**Figure 8:**
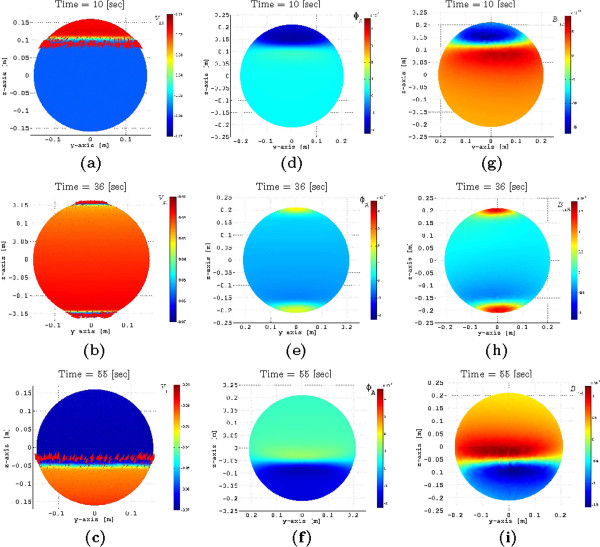
**FEM solution at time instants t*=*10 [s], 36 [s], 55 [s] for one pacemaker on the fundus of a spherical myometrium, assuming anisotropy.** (**a**)-(**c**) transmembrane potential and source current density distribution at the myometrium, (**d**)-(**f**) electrical potential at the abdominal surface, and (**g**)-(**i**) magnetic field density at the abdominal surface.

Figure 8 shows several snapshots of the FEM solution for one pacemaker on the fundus of a spherical myometrium, assuming anisotropy as illustrated in Figure 7. Figure 8 (a)-(c) illustrates the transmembrane potential and source current density distribution at the myometrium, Figure 8 (d)-(f) shows the electrical potential at the abdominal surface, and Figure 8 (g)-(i) illustrates the magnetic field density at the abdominal surface. The magnetic field measured at the abdominal surface, ***B***_MMG_ (the magnetomyogram field), is proportional to $B_{n_{A}},$ the projection of ***B*** onto the normal vector of the abdominal surface, $n_{A}.$

**Figure 9:**
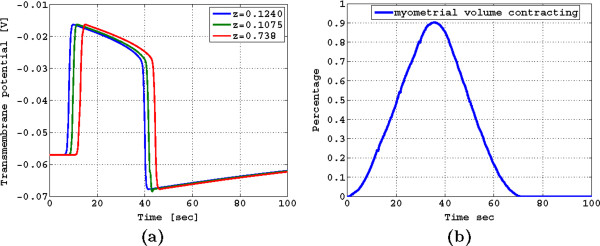
(a) Temporal response of the FEM solutions for transmembrane potential at different elevations; (b) Percentage of contracting myometrial volume as a function of time.

In Figure 9 (a) we illustrate the temporal response of the FEM solutions for the transmembrane potential at different elevations over time. It can be seen that a stable traveling waveform has been established as the shape remains the same. Also, the maximum depolarization is −16 mV, the average potential in the plateau area is −25 mV, and the transmembrane potential duration, before hyperpolarization, is approximately 35 s, which are fair approximations with respect to the average recorded transmembrane potentials discussed in [13,30]. Note that our ionic current model introduces hyperpolarization, which constrains the excitability of the cell, and thus consecutive contractions can only take place until *v*_m_reaches resting potential. In our case, our model can simulate a minimum period of one contraction every 240 s, allowing us to model labor contractions whose period reduces to about one contraction every 300s.

In Figure 9(b) we illustrate the percentage of the contracting myometrial volume as function of time, which in [7,9] was used as a reference to compute the changes in the intrauterine pressure due to a contraction. Interestingly, we observe that the percentage of the contracting myometrial volume as a function of time has the symmetric properties and time duration of the intrauterine pressure waveforms of a pregnant human myometrium at term, as discussed in [9].

## Discussion

Our numerical simulation results (see Figure 8) show that, because of the anisotropy in the conductivity, the direction of the current density ***J***_s_is rotated at a certain angle from the main direction of the propagating transmembrane potential *v*_m_, and it is the transversal component of this current, parallel to the x-y plane, which generates the magnetic field $B_{n_{A}}.$ This observation is in agreement with the analysis presented in [37], and it is important to take it into account when interpreting the magnetic field measurements generated by uterine contractions in the presence of volume conductor geometry. Therefore, the spatial signature of $B_{n_{A}}$ is highly dependent on the fiber orientation of the myometrium. Because of the proximity of the sensors and the myometrium, it is not strictly applicable to assume a moving dipole parallel to the direction of propagation of the transmembrane potential as the main model for the current source generating the measured magnetic field. This last interpretation might be suitable in the case where the transverse length of the transmembrane potential front is short in comparison to the area covered by the array of sensors. In contrast, if the transverse length is larger and thus not covered by the measured area, (for example, when several cells are recruited), then it is suitable to consider a moving line source (stretched ring) model instead.

Though our volume conductor geometry is an oversimplification of a real anatomical structure, certain aspects of it stand as a fair approximation of the volume conductor geometry when performing MMG measurements. For example, the SARA system, known as the superconducting quantum interference device array for reproductive assessment ^a^(SQUID Array for Reproductive Assessment) is a passive, stationary, floor-mounted instrument at which the patient sits and leans her abdomen against a concave surface which contains an array of 151 sensors. The effect of leaning the abdomen on the concave surface works as a way to standardize the abdominal surface making the spherical model very suitable to represent the measuring surface. Also, a spherical uterus is good approximation at the early stage of pregnancy but not necessarily through all stages. In this sense, a more realistic population-average uterine model should be constructed from magnetic resonance images. Unfortunately, exposing of pregnant patients to MRI is not currently the norm, and thus having access to average uterine geometry for different stages of pregnancy is not yet possible.

The ionic current model based on extended FHN equations can reproduce a good approximation of the plateau-type transmembrane potential recorded in human myocytes, however, it introduces hyperpolarization, which constrains the excitability of the cell, and thus consecutive contractions can only take place until *v*_m_ reaches resting potential. In our case, our model can simulate a minimum period of one contraction every 240 s, allowing us to model labor contractions whose period reduces to about one contraction every 300 s. In addition, note that a larger *ε*_2_ value can extend the transmembrane potential duration to values closer to the average duration reported in [13,30]. However, a larger *ε*_2_value also extends the duration of hyperpolarization and therefore the plateau of the curve describing the percentage of contracting myometrial volume. This last observation is the result of the combined effect of the transmembrane potential duration and the geometry of the uterus. Clinical observations on the shape of the uterine pressure wave have found correlation between the rising slope of the waveform and contraction efficiency [38], namely, the steeper the slope the more efficient the contraction is. Additional numerical examples, using one pacemaker at the same position, show that our model is consistent with the latter observation, since we found that changing the propagation speed of the activity modifies the shape of the percentage of the contracting myometrial volume as a function of time. In particular, a faster speed of propagation of the main transmembrane potential front increases the rising slope the percentage of the contracting myometrial volume, however, above a certain threshold it reduces the duration of the contraction. Placing two pacemakers at each extreme of the spherical domain, one at the fundus and the other one at the cervix, doubles the rising slope of the percentage of the contracting myometrial volume curve, and, depending on the duration of the transmembrane potential, it introduces a plateau in the curve, which implies that all volume is contracting. The symmetry of the contracting myometrial volume curve generated using our model is primarily due to the symmetry of the volume conductor used in our simulations and the duration of the transmembrane potential simulated.

Modeling the different stages of pregnancies can be accommodated using this model. First the volume conductor geometry should be scaled to the average volume size corresponding to stage of pregnancy of interest. Specifically, the fetal, the uterine, and the abdominal shapes should be modified accordingly using reported values in the literature (e.g., see [3] for uterine shapes). Second, the conductivity values of the volume conductor model should be set with respect to the stage of pregnancy. For example, the conductivity of the intrauterine cavity given by the amniotic fluid is known to change as the pregnancy develops (e.g., see [36]). Regarding the equal anisotropy ratio, this can be computed by using the reported values of the speed of propagation, the resting transmembrane potential, and the updated ionic current parameters such these fit the transmembrane potential for the specific stage. Third, the boundary conditions remain the same across all periods, noting that for boundary condition given by Eq. (46), *λ* should be set to 1 or 0 depending on the presence of vernix caseosa. Fourth, incorporation of a bursting type transmembrane potential can be done using the FHN model by adding a periodic function to Eq. (13), which control the excitability of the cell. However, more realistic and complex shapes of transmembrane potential can be designed by incorporating, for example, the ionic current model presented in [17].

Additional approaches to validate our mathematical model should consider comparisons against real magnetic and potential field measurements at the level of the abdomen, which leads automatically to the next natural question: how to solve the inverse problem of our model? Explicitly, given a set of measurements and a parametric model, can we infer the value of certain parameters of interest, such as the number of stimui and their positions, the conductivity tensor in the domain, initial and boundary conditions, etc? Solving the inverse problem stands as the connecting bridge between multiscale modeling of the pregnant uterus and clinical applications. In particular, as a first approach we are planning to use MMG measurements to estimate the current density in the myometrium and thus search for features that characterize contractions patterns which lead to preterm-labor.

## Conclusion

We proposed a multiscale-forward electromagnetic model of uterine contractions during pregnancy. Our model incorporates knowledge of the electrophysiological aspects of uterine contractions during pregnancy at both the cellular and organ levels. We applied a bidomain approach for modeling the propagation of the myometrium transmembrane potential *v*_m_ on the uterus and used this to compute the action potential *ϕ*and the magnetic field ***B***at the abdominal surface. We introduced a modified version of the FitzHugh-Nagumo equation for modeling the ionic currents in each cell. Though our ionic current model does not consider **Ca**^2 + ^dynamics explicitly, the simplicity of the modified equation allows for the propagating action potential to be modeled under well defined conditions as shown in this paper. We also proposed a general approach to design conductivity tensors in the myometrium and to estimate the conductivity tensor values in the extracellular and intracellular domains. We introduced a simplified geometry for the problem and proposed a discretized model solution based on a finite element method approach. Finally, we illustrated our modeling approach through a numerical example by modeling uterine contractions at term. Our model is potentially important as a tool for helping characterize contractions and for predicting labor using MMG and EMG.

As part of our future work, we will investigate pear-shaped uterine domains as a way to approximate better the uterine geometry. We will also include more accurate ionic current models as in [13-15,17] and will consider spatial variations of the fiber orientation.

## Endnote

^a^SARA was built in collaboration with VSMMedTech Ltd., Canada and is installed at the University of Arkansas for Medical Sciences (UAMS) Hospital.

## Competing interests

The authors declare that they have no competing interests.

## Authors’ contributions

PSL contributed to the model design, implemented the model, and drafted the manuscript. HE and HP contributed to the model design and guided the model validation. AN coordinated the study, contributed to the model design, and helped in preparing the draft the manuscript. All authors read and approved the final manuscript.

## Pre-publication history

The pre-publication history for this paper can be accessed here:

http://www.biomedcentral.com/1756-6649/12/4/prepub
